# Robust meta-analysis of gene expression using the elastic net

**DOI:** 10.1093/nar/gkv229

**Published:** 2015-03-31

**Authors:** Jacob J. Hughey, Atul J. Butte

**Affiliations:** Division of Systems Medicine, Department of Pediatrics, Department of Pediatrics, Stanford University, Stanford, CA 94305, USA

## Abstract

Meta-analysis of gene expression has enabled numerous insights into biological systems, but current methods have several limitations. We developed a method to perform a meta-analysis using the elastic net, a powerful and versatile approach for classification and regression. To demonstrate the utility of our method, we conducted a meta-analysis of lung cancer gene expression based on publicly available data. Using 629 samples from five data sets, we trained a multinomial classifier to distinguish between four lung cancer subtypes. Our meta-analysis-derived classifier included 58 genes and achieved 91% accuracy on leave-one-study-out cross-validation and on three independent data sets. Our method makes meta-analysis of gene expression more systematic and expands the range of questions that a meta-analysis can be used to address. As the amount of publicly available gene expression data continues to grow, our method will be an effective tool to help distill these data into knowledge.

## INTRODUCTION

The amount and continued growth of publicly available gene expression data are staggering. NCBI GEO and ArrayExpress currently have available more than 1.6 million samples spread across more than 54 000 studies (1,2). To take advantage of all these data, meta-analysis of gene expression has become an important tool (3). By combining multiple data sets, a meta-analysis can gain statistical power and overcome the biases of individual studies (4). Meta-analysis of gene expression has been used to uncover disease subtypes (5), to predict survival (6) and to discover biomarkers and therapeutic targets (7–9).

Despite those successes, current methods for meta-analysis of gene expression have several limitations. Often the goal of a meta-analysis is to obtain a small set of genes whose expression correlates with the variable of interest, such as healthy versus disease. Most current methods select genes based on univariate summary statistics, such as *P*-value of differential expression. As a result, current methods struggle to select genes that each contribute non-redundant information and to systematically determine (e.g. with cross-validation) how many genes to include in the set. Such methods are also difficult to generalize when comparing more than two conditions. Finally, meta-analyses whose goal is diagnosis or prognosis often need to account for additional variables, such as histological findings or patient characteristics. Unfortunately, incorporating covariates into a meta-analysis of gene expression is a problem that currently has no general solution.

The elastic net (10), a generalization of ridge regression (11) and the lasso (12), is a powerful and versatile method for classification and regression. The elastic net is a regularization method for fitting a generalized linear model. Because the elastic net builds a multivariate predictive model, it is amenable to cross-validation and can easily assimilate continuous and categorical features. In addition, the elastic net can perform feature selection, which means the resulting model can include as few features as desired. The elastic net has found use in numerous and diverse applications, including identification of genomic markers of drug sensitivity (13), development of a predictor of age based on DNA methylation (14) and identification of risk factors for binge drinking (15). The elastic net is particularly well suited to genome-scale data, which typically has many more features than observations. Despite its power and versatility, however, the elastic net has not been applied to meta-analysis.

In this work, we describe a methodological framework for using the elastic net to perform a meta-analysis of gene expression. To show how our approach addresses the limitations of previous methods, we perform a meta-analysis of lung cancer gene expression based on publicly available data. Our meta-analysis results in a robust and accurate multinomial classifier that distinguishes between four lung cancer subtypes using a small set of genes. Our method also enables us to rigorously demonstrate the value of a meta-analysis, in that training a classifier on multiple studies improves prediction compared to training a classifier on only one study.

## MATERIALS AND METHODS

### Meta-analysis of lung cancer gene expression

We curated data from eight publicly available microarray studies of lung cancer (Supplementary Table S1). For each study, we used the information regarding each sample's cancer subtype, as well as any information regarding patient sex, age and smoking status, as provided. For GSE11969, smoking history was converted from Brinkman index (number of cigarettes per day multiplied by number of years of smoking) to either ‘current’ (if Brinkman index was greater than zero) or ‘never’ otherwise. For visualizing the samples using t-SNE, we used samples corresponding to cancer subtypes that were present in at least two studies. For our meta-analysis, we used only the samples in each data set that were histologically defined as adenocarcinoma (AD), squamous cell carcinoma (SQ), small cell lung carcinoma (SCLC), or carcinoid (CAR). The discovery data sets were selected to include a variety of microarray platforms and to have a sufficient number of samples of SCLC and CAR. Because the Bhattacharjee data set is very heavily biased toward AD, but also has samples from the other subtypes, we included only 60 of the AD samples. Altogether, the merged discovery data comprised 639 samples and 7200 genes that were present in all five discovery data sets. We used *α* = 0.9 for the elastic net penalty, where *α* = 0 corresponds to ridge (L2-norm penalty) and *α* = 1 corresponds to lasso (L1-norm penalty). Lower values of *α* led to a classifier with more genes, but with identical performance. We always set glmnet's *intercept* option to *true*, although setting it to *false* did not appreciably affect the results. When calculating accuracy, the predicted class for each sample was taken to be the class with the highest probability.

### Processing each data set

Each data set should first be curated to include the samples of interest and any information for each sample that the classifier will use, such as cancer subtype. Expression values in each data set are normalized and log-transformed (or equivalent). Raw Affymetrix data are normalized using RMA (16) and mapped to Entrez Gene IDs using custom CDFs (17). If raw data are not available, processed GEO data are fetched using GEOquery (18) and microarray probes are mapped to Entrez Gene IDs (R package org.Hs.eg.db for human genes). For processed data, if multiple probes map to the same Entrez Gene ID, the expression value for that Entrez Gene ID is calculated as the median of the expression values of those probes. Missing expression values (for genes whose expression is present for some samples and not others in the data set) are imputed using nearest neighbor imputation (R package impute).

### Merging data sets

In our framework for meta-analysis, first the data sets are merged, then the analysis is done (19). One challenge with a meta-analysis of gene expression is that each data set may have expression values for a slightly different set of genes. In order to use the elastic net, the expression data are reduced to the set of genes that are common to all data sets being merged. Each data set is then globally scaled (across all genes and samples) to have mean 0 and standard deviation 1, a step which we have found improves the robustness of ComBat's cross-study normalization. ComBat, an empirical Bayes method, is then used to perform cross-study normalization (20). Importantly, our cross-study normalization does not use the sample metadata (e.g. cancer subtype). Because the goal of our method is to predict that information, it must be treated as unknown in the merging step.

### Using the elastic net

When we refer to the ‘elastic net,’ we mean the method of using the elastic net penalty to fit a generalized linear model (GLM), as implemented in the R package glmnet (21). Before the merged gene expression data are passed to glmnet, the values for each gene are centered to zero. Glmnet's *standardize* option is then set to *false*. If the distribution of classes (e.g. types of cancer) in the training set is representative of the expected distribution in the testing set, i.e. they are an accurate prior, then glmnet's *intercept* option can be set to *true*. If desired, additional variables can be added alongside the genes. Categorical variables can be incorporated as dummy variables. Continuous variables should be scaled to have the same mean and standard deviation as the gene expression data. By default, when training the classifier, the samples are weighted such that each study is weighted equally, although this can be adjusted.

The objective function of the elastic net takes the form of ‘loss + penalty’:

\begin{equation*} \mathop {\min }\limits_{\beta _0 ,\beta } \frac{1}{N}\sum\limits_{i = 1}^N {w_i l(y_i ,\beta _0 + \beta ^T x_i ) + \lambda \left( {(1 - \alpha )||\beta ||_2^2 /2 + \alpha ||\beta ||_1 } \right),} \end{equation*}

where *β*_0_ and *β* are the coefficients of the GLM, *N* is the number of observations (i.e. samples), *w_i_* is the weight of observation *i, l*(*y*,*η*) is the negative log-likelihood contribution for observation *i* (the functional form of *l* depends on the type of model being fit), *λ* is the regularization parameter (which controls the amount of shrinkage), *α* is the elastic net penalty (which controls the balance between ridge and lasso regression), ∥*β*∥_2_ is the L2-norm of *β* and ∥*β*∥_1_ is the L1-norm of *β*. The weights are scaled such that

\begin{equation*} \sum\limits_{i = 1}^N {w_i = N.} \end{equation*}

If there are *M* batches (i.e. studies), then to achieve equal weighting of each batch, we set

\begin{equation*} w_i = \frac{M}{{n_i}}, \end{equation*}

where *n_i_* is the number of observations in the batch to which observation *i* belongs.

### Cross-validation of elastic net classifier

After merging the discovery data sets, cross-validation is performed to determine how the performance of the classifier depends on the regularization parameter of the elastic net. By default, our method uses leave-one-study-out cross-validation, although random n-fold cross-validation is also possible. Using leave-one-study-out cross-validation requires that each class has samples from at least two studies.

### Validating the classifier on independent data sets

Our procedure for validation is designed so that each validation data set is tested independently of the others. For each validation data set, all discovery data sets and the current validation data set are merged in the manner described above. Note that the set of genes in this merged data set may be slightly smaller than the set of genes in the merged discovery data set that was used for cross-validation. In particular, a gene in the classifier from cross-validation might not have been measured in the current validation data set. In addition, the inclusion of the validation data set may slightly alter the cross-study-normalized expression values. Therefore, after merging the data sets, a new classifier is trained on the samples from the discovery data sets using the regularization parameter obtained from previous leave-one-study-out cross-validation. Again, samples from the discovery data sets are weighted such that each study is weighted equally. The classifier is then tested on the samples from the current validation data set. The process is repeated for each validation data set.

## RESULTS

### Approach for performing meta-analysis using the elastic net

**Figure 1.**
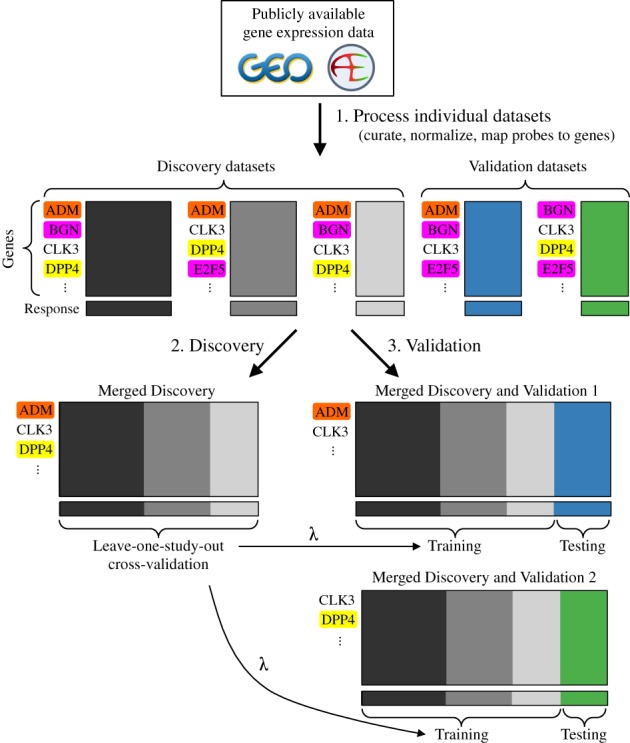
Workflow for performing a meta-analysis of gene expression using the elastic net. In example, three data sets are used for discovery and two are used for validation. In each data set, genes are in rows and samples are in columns. Genes not measured on every data set being merged are removed. The genes highlighted in magenta were not measured on every discovery data set. The gene highlighted in orange was measured on every discovery data set and on validation data set 1, but not on validation data set 2. The gene highlighted in yellow was measured on every discovery data set and on validation data set 2, but not on validation data set 1. After data sets are merged, batch effects are corrected using ComBat. The optimal value of the regularization parameter *λ* obtained from cross-validation is used to train the models in the validation phase.

Our approach can be divided into three stages (Figure 1). In the first stage, each data set is processed individually, which includes normalization and mapping probes to Entrez Gene IDs. In the second stage, discovery data sets are merged and the elastic net is used to perform cross-validation. One of the results of cross-validation is a value for the regularization parameter lambda, which determines how much shrinkage is used to train the model. In the third stage, each validation data set is individually merged with the discovery data sets, the elastic net and the pre-determined value of lambda are used to train a model on the discovery samples, then the model is tested on the validation samples. At every step in which data sets are merged, only Entrez Gene IDs measured on every data set being merged are included, and cross-study normalization is performed using ComBat (20).

To demonstrate the utility of our method, we conducted a meta-analysis of lung cancer gene expression using publicly available data. Our goal was to use multiple studies to build a robust multinomial classifier containing a small set of genes that could distinguish between several lung cancer subtypes. Such a classifier and corresponding gene set could aid development of better diagnostic tools and could inform our understanding of the biology of the respective subtypes, but could not be built using existing methods for meta-analysis.

### Unsupervised analysis of lung cancer subtypes

**Figure 2.**
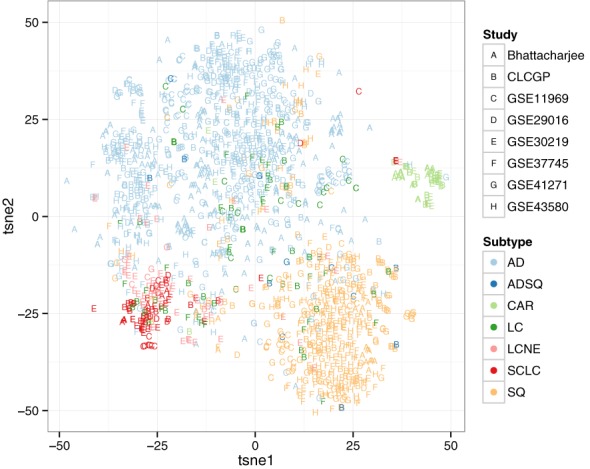
t-SNE plot of gene expression in lung cancer samples from eight publicly available data sets. Data sets were merged as described in Methods. Each point corresponds to one sample. The letter corresponds to the data set. The color corresponds to the subtype: adenocarcinoma (AD), adenosquamous (ADSQ), carcinoid (CAR), large cell carcinoma (LC), large cell neuroendocrine carcinoma (LCNE), small cell lung carcinoma (SCLC), or squamous (SQ).

We first curated eight publicly available data sets containing samples of various lung cancer subtypes (Supplementary Table S1 and (22–29)). We then analyzed the data in an unsupervised manner, to verify that samples of the same subtype clustered together, i.e. the existing subtypes are appropriate for classification. Samples from all eight data sets were merged and visualized using the non-linear dimensionality reduction algorithm called t-SNE, which excels at revealing the structure of high-dimensional data sets (30). Based on the results of t-SNE (Figure 2), we chose to use four subtypes for our multinomial classifier: AD, SQ, SCLC and CAR. We selected five of the eight data sets for discovery and three for validation, such that each of the four subtypes was represented in at least two discovery data sets. Due to the limited number of data sets with samples for SCLC and CAR, however, only two of the five discovery data sets contained samples from all four subtypes (Supplementary Table S1).

### Training the multinomial classifier based on multiple data sets

**Figure 3.**
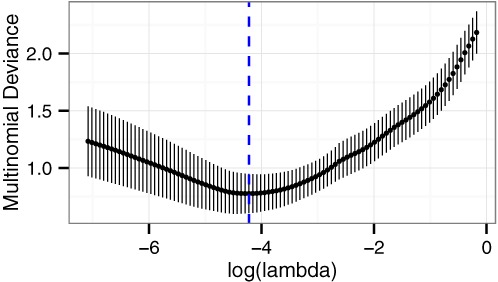
Multinomial deviance as a function of the regularization parameter lambda for leave-one-study-out cross-validation on the discovery data sets. Points correspond to the mean, error bars correspond to the standard deviation. The blue dashed line marks the value of lambda at which the multinomial deviance is at a minimum.

We next merged only the discovery data sets and used the elastic net to perform leave-one-study-out cross-validation across a range of values of the regularization parameter lambda (Figure 3 and Supplementary Figure S1). As the regularization parameter increases, the elastic net imposes more shrinkage on the coefficients of the model, resulting in a model with fewer features (in this case, genes). For training a multinomial classifier, an appropriate loss function is the multinomial deviance (31). Importantly, the multinomial deviance did not monotonically decrease as the regularization parameter decreased. This result implies that there is an optimal number of genes to include in the classifier, and including more genes beyond that optimum actually worsens the classifier's performance.

**Figure 4.**
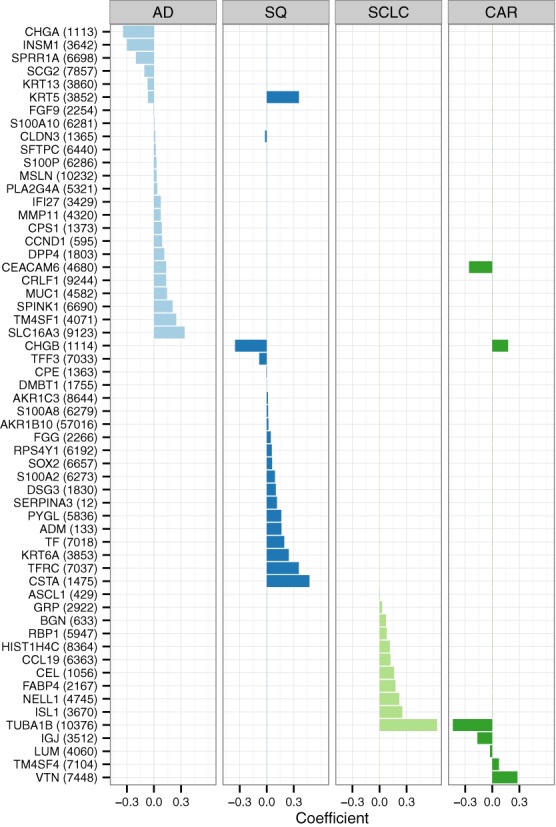
Selected genes and their coefficients in the multinomial classifier. The classifier was trained on the discovery data sets using the regularization parameter that gave the lowest multinomial deviance on leave-one-study-out cross-validation. Only genes with non-zero coefficients for at least one subtype are shown. Entrez Gene ID shown in parentheses. A positive coefficient for a particular gene and subtype indicates that increased expression of that gene increases the probability that a sample belongs to that subtype.

At the value of the regularization parameter that gave the lowest multinomial deviance, the overall accuracy (fraction of correctly classified samples) of the multinomial classifier on cross-validation was 91.2% (Supplementary Figure S2, Supplementary Table S2). Prediction accuracies for the four cancer subtypes ranged from 74% (SCLC) to 97% (AD). Using that value of the regularization parameter, we trained a classifier on all samples from the discovery data sets. The resulting classifier contained 58 genes (Figure 4). Similar to previous work using regularization to build a multinomial classifier (32), the genes with non-zero coefficients for each subtype are almost mutually exclusive. In support of our methodology, the expression of the 58 genes differed between subtypes across the multiple discovery data sets (Supplementary Figure S3).

### Successful validation on independent data sets

**Figure 5.**
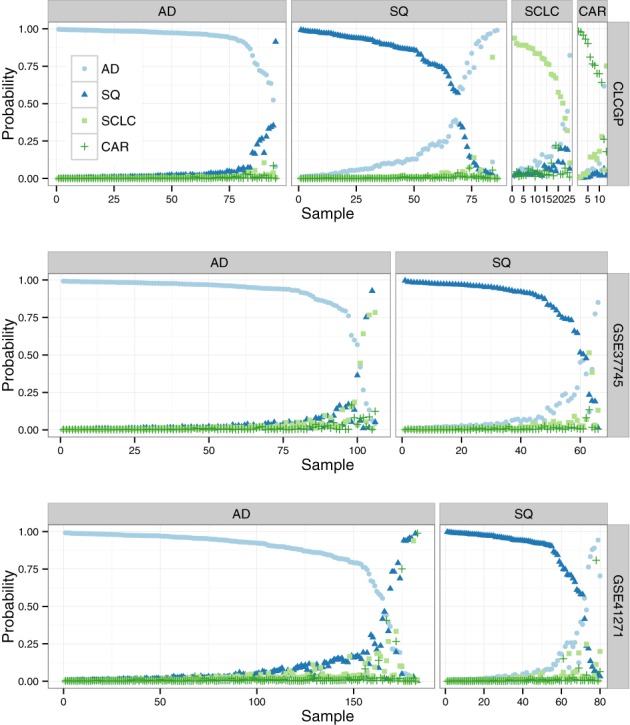
Estimated probabilities for samples in validation data sets. The classifier was trained on the discovery data sets (merged with the respective validation data set) using the regularization parameter that gave the lowest multinomial deviance on leave-one-study-out cross-validation. For each sample, there are four points, corresponding to the probability that the sample belongs to the respective subtype. Within each data set, samples are partitioned by their true subtype. Within each data set and subtype, samples are sorted by the probability of the true subtype. For most samples, the probability of the true subtype is near 1, indicating unambiguous classification.

To further test our method, we validated the classifier on three independent data sets (Figure 5). Our approach for validation accounts for the possibility that a gene might have been measured in the discovery data sets, but not in a particular validation data set (Figure 1). Across the three validation data sets, the overall accuracy was 91.3% (Supplementary Table S3), nearly identical to that obtained during cross-validation. The accuracy of our classifier is similar to that reported for a microRNA-based diagnostic assay targeting the same four lung cancer subtypes (33). These results indicate that our method can successfully extract a robust signal from gene expression data derived from multiple studies. Furthermore, our approach can produce an accurate multinomial classifier, even if not all discovery data sets have samples from all classes.

### Combining discovery data sets improves classifier performance

We compared the performance of the classifier trained on all the discovery data sets against that of classifiers trained on only GSE30219 or only the Bhattacharjee data set (the two discovery data sets with samples from all four subtypes). For each of the two classifiers, the value of the regularization parameter was determined by 5-fold cross-validation on the samples of the respective data set. For the classifier trained on GSE30219, the overall accuracy on the validation data sets was 87.8%, only marginally worse than the performance of the classifier trained on all the discovery data sets (Supplementary Table S4). For the classifier trained on the Bhattacharjee data set, however, the overall accuracy on the validation data sets dropped to 76.1% (Supplementary Table S5). These findings exemplify the power of a meta-analysis to overcome the biases of individual studies.

### The elastic net outperforms the method of nearest shrunken centroids

Although our approach to build a predictive model based on multiple data sets uses the elastic net, it can also be adapted to use other machine learning techniques. Within the context of our lung cancer meta-analysis, we compared the elastic net to the method of nearest shrunken centroids called PAM (32). Similar to the elastic net, PAM uses regularization, but PAM selects genes in a univariate manner. When restricted to a classifier of approximately 100 genes, PAM performed comparably to the elastic net for three of the four subtypes (Supplementary Table S6). For SCLC, however, PAM required several thousand genes in order to build a classifier approximately as accurate as the one based on the elastic net (Supplementary Table S7). This disparity in performance suggests that the multivariate model trained using the elastic net is superior at efficiently extracting signal from genome-scale data.

### Straightforward incorporation of additional features alongside the genes

When the goal of a meta-analysis of gene expression is to build a predictive model for diagnosis or prognosis, it is important to account for other possibly predictive variables in addition to gene expression. Because our method merges multiple data sets into a single matrix, additional variables, such as patient characteristics, can simply be appended as new features in the matrix. The elastic net, which can handle both continuous and categorical features, can then build a predictive model based on gene expression and the additional variables.

**Figure 6.**
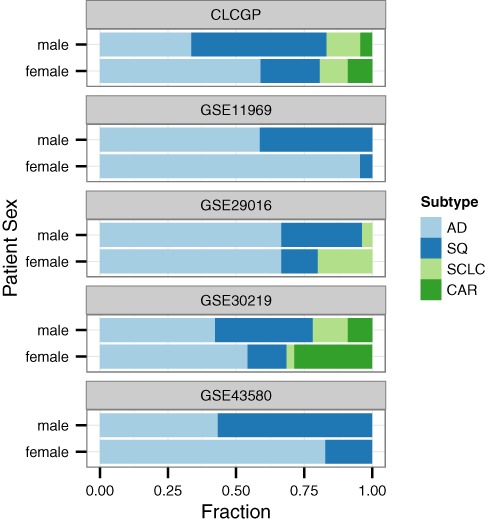
Relative fraction of each of the four lung cancer subtypes across five data sets, stratified by patient sex. For each data set, samples not from one of the four subtypes were excluded.

To demonstrate the feasibility of including variables using our method, we performed a second meta-analysis of the four lung cancer subtypes. Samples from five data sets included patient sex, age and smoking status (current, former, or never). Using those data sets, we performed leave-one-study-out cross-validation with and without those three variables. We expected that at least patient sex would be included in the classifier and would improve prediction, because men and women have different distributions of lung cancer subtypes. In particular, men have a lower relative frequency of AD and a higher relative frequency of SQ (Figure 6 and (34)). Surprisingly, however, patient sex, age and smoking status did not improve the classifier (Supplementary Figure S4). In fact, at the optimal value of the regularization parameter, the classifier did not include any of the three variables (Supplementary Figure S5).

**Figure 7.**
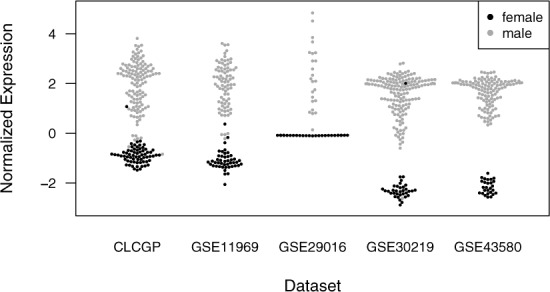
Normalized expression of RPS4Y1 across five data sets. Each point corresponds to a sample. The color corresponds to the sex of the patient for that sample. The expression data were first normalized within each study, then normalized across studies using ComBat. For ease of visualization, one sample from GSE29016 that had a normalized expression value of 8.3 (and was from a male patient) has been omitted. Several samples appear to have incorrect information for patient sex.

We hypothesized that any information contained in the patient variables might already be present in the gene expression. We noticed that one of the genes in the classifier (Figure 4 and Supplementary Figure S5), RPS4Y1, is on the Y chromosome (and not on the X chromosome). In the classifier, RPS4Y1 had a positive coefficient for SQ, meaning that higher expression of RPS4Y1 increased the probability that a sample would be classified as SQ. Expression of RPS4Y1 was strongly correlated with patient sex and was higher in males (Figure 7). In fact, of all the genes on the Y chromosome whose expression was measured on each data set, RPS4Y1 had by far the largest difference in expression between males and females (Supplementary Figure S6). We reasoned that expression of RPS4Y1 was serving as a proxy for patient sex. When we excluded from the meta-analysis all genes on the Y chromosome, the elastic net selected patient sex as a feature in the classifier, with a coefficient such that a sample from a male would be given a higher probability of SQ than a sample from a female (Supplementary Figure S7). Thus, our method enables one to smoothly assimilate additional variables into a meta-analysis of gene expression and to evaluate whether the additional variables improve prediction.

## DISCUSSION

Our methodological framework represents a significant advance in meta-analysis of gene expression. Rather than analyzing each data set separately and then combining summary statistics, our method performs cross-study normalization to merge the raw data and then analyzes the merged data using the elastic net. By expanding the reach of the elastic net to analyze multiple studies, our method offers several advantages compared to previous methods, including those that directly merge the raw data (35,36). Most importantly, the elastic net builds a multivariate, predictive model and performs feature selection. As a result, one can use cross-validation to systematically determine how many and which genes belong in the ‘expression signature’ of the condition(s) of interest. As we demonstrate in our meta-analysis of lung cancer subtypes, the elastic net enables meta-analysis of more than two discrete conditions. Because the elastic net can be applied to several types of regression problems, our approach also makes possible meta-analysis of continuous variables, such as survival time. Furthermore, the elastic net makes it straightforward to incorporate additional variables alongside the gene expression, which can reveal when a gene's expression is related to a covariate and not strictly to the biology of interest.

The prediction accuracy of our multinomial classifier varied considerably across the four subtypes. Several factors could explain this behavior, in particular the relatively high misclassification rate for SCLC. The first is the small sample size for SCLC. Other potential factors include imperfect correction of batch effects and an inconsistent definition of SCLC across studies. In addition, our unsupervised analysis suggests that although most samples of SCLC cluster together, a number of AD and SQ samples have a pattern of gene expression that is similar to SCLC (Figure 2). This may indicate heterogeneity within AD and SQ, misdiagnosis, or both. Although CAR has a similar number of samples as SCLC, the gene expression of CAR seems to be quite distinct from that of the other subtypes (Figure 2), which explains the higher prediction accuracy for CAR compared to SCLC.

Even if prediction is not the primary goal of a meta-analysis, our approach is useful in generating a prioritized list of genes for further investigation. Many of the 58 genes in our meta-analysis-derived classifier of lung cancer subtypes are known to be relevant to lung cancer. For example, expression of MUC1 is associated with patient outcome (37) and MUC1 is being pursued as a therapeutic target in both breast and lung cancer (38). Keratins, three of which are among the 58 genes (KRT5, KRT6A and KRT13), are reliable immunohistochemical markers of SQ (39). A number of genes in the classifier were also identified in previous analyses of lung cancer subtypes (22,40).

Batch effects are always a concern in biological data. Our method corrects for batch effects and performs cross-study normalization using ComBat. Although ComBat has worked well in our hands, one should be especially cautious when merging data sets that are extremely unbalanced, e.g. when one data set has samples of one disease subtype and a second data set has samples of a different subtype. In that case, neither ComBat nor any other algorithm can reliably distinguish batch effects from real differential expression. Whereas our method uses ComBat because the batch information is known, a method called frozen surrogate variable analysis (fSVA) is designed for prediction when the batch information is unknown (41). In the future, it may be possible to use our approach to train a predictive model based on multiple data sets, then use fSVA to predict the class or outcome of new individual samples.

One caveat with our approach is that the microarray probes in each data set are mapped to Entrez Gene IDs and their expression is condensed to one value per Entrez Gene ID, before the data are analyzed. This step is necessary, in order to merge the data sets. The cost of this step, however, is that if one probe for a gene is differentially expressed but other probes for the gene are not, the signal from the one probe could be drowned out by the noise of the others.

In the current implementation of our approach, the step of merging data sets involves excluding any gene not measured on each data set. Using only the intersection of genes may at first seem to be a severe restriction, especially as the number of data sets increases. The size of the intersection, however, is determined not by the total number of data sets, but by the number of unique microarray platforms. Given that the vast majority of publicly available gene expression data are based on a small number of platforms and that any given phenotype is typically associated with the expression of many genes, we believe that the current implementation will work well for most meta-analyses. In this respect, our deliberate choice to use the Bhattacharjee data set for discovery represents a near worst-case scenario. Excluding the Bhattacharjee data set, which was collected on the Affymetrix HGU95Av2 GeneChip, would raise the number of Entrez Gene IDs in the merged discovery data from 7200 to 13 609 (at the cost of losing valuable samples for SCLC and CAR). In the future, our method could be altered to impute the expression of genes that are present in some data sets but absent from others. Such imputation would be unlikely to significantly improve prediction, but would allow those genes to be included in the predictive model.

Our approach both addresses the limitations of previous methods and expands the range of questions that can be addressed using meta-analysis of gene expression. As the amount of publicly available gene expression data continues to grow, our method will be an effective tool to help distill these data into knowledge.

## AVAILABILITY

Software and instructions for performing a meta-analysis, as well as all data necessary to reproduce our meta-analysis of lung cancer, are available at https://zenodo.org/record/16006. All computation is done in R.

## SUPPLEMENTARY DATA

Supplementary Data are available at NAR Online.

SUPPLEMENTARY DATA
