# Supplementary material for: Robust meta-analysis of gene expression using the elastic net
Source: Nucleic Acids Res. 2015 Mar 31;43(12):e79. doi: 10.1093/nar/gkv229 (PMC4499117; doi:10.1093/nar/gkv229)
Supplement: SUPPLEMENTARY DATA [file supp_43_12_e79__index.html]

Robust meta-analysis of gene expression using the elastic net — SUPPLEMENTARY DATA 

# Robust meta-analysis of gene expression using the elastic net

## SUPPLEMENTARY DATA

**Files in this Data Supplement:**

- Supplementary Figures
- Supplementary Tables
