# Supplementary material for: Robust meta-analysis of gene expression using the elastic net
Source: Nucleic Acids Res. 2015 Mar 31;43(12):e79. doi: 10.1093/nar/gkv229 (PMC4499117; doi:10.1093/nar/gkv229)
Supplement: SUPPLEMENTARY DATA [file supp_gkv229_nar-00280-met-n-2015-File010.pdf]

Supplementary Material: Robust meta-analysis of gene expression using the elastic net

Jacob J. Hughey[1] and Atul J. Butte[*,1]

[1]Department of Pediatrics, Stanford University, Stanford, CA 94305, US

[*]To whom correspondence should be addressed. Tel: +1 650 725 1337;
Fax: +1 650 723 7070; Email: abutte@stanford.edu.

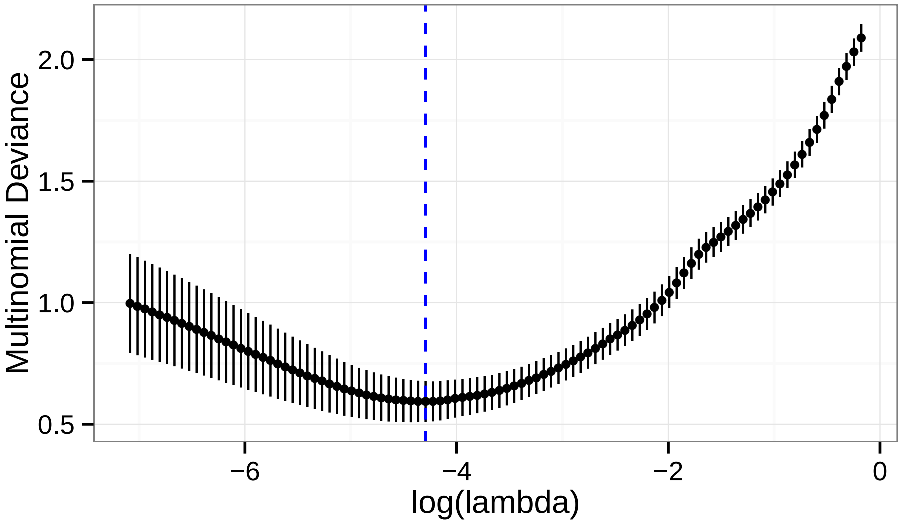

Supplementary Figure S1: Multinomial deviance as a function of the regularization parameter lambda for 10-fold cross-validation on the samples in the discovery datasets. Points correspond to the mean, error bars correspond to the standard deviation. The blue dashed line marks the value of lambda at which the multinomial deviance is at a minimum. The optimum values of lambda for leave-one-study-out (Figure 3) and 10-fold are very similar, indicating that the resulting classifiers and performance on validation datasets would also be very similar. At the optimum value of lambda, however, 10-fold cross-validation gives an overly optimistic value for the multinomial deviance.

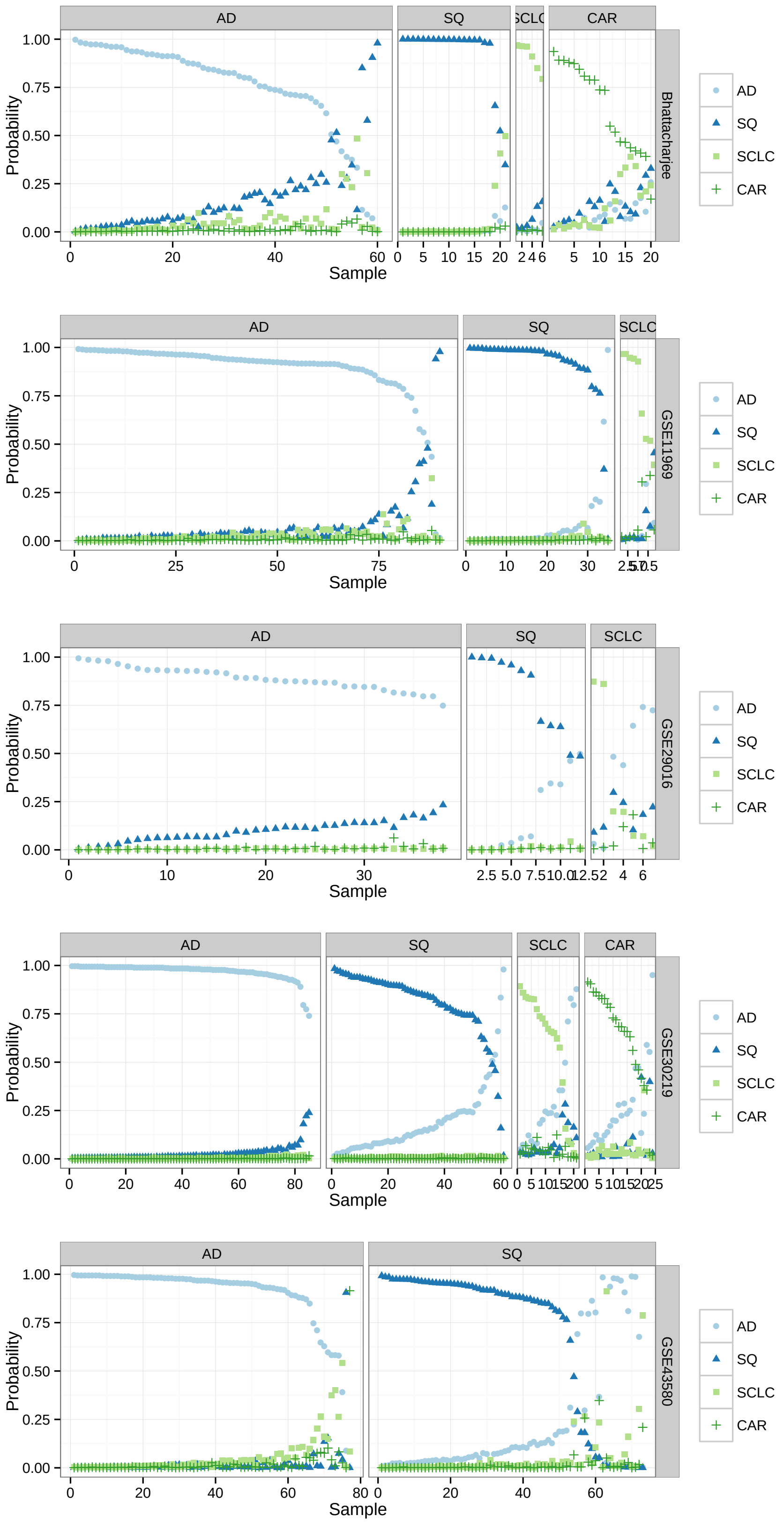

Supplementary Figure S2: Estimated probabilities for samples in discovery datasets on cross-validation. For each sample, there are four points, corresponding to the probability that the sample belongs to the respective class. Within each dataset, samples are partitioned by their true class. Within each dataset and class, samples are sorted by the probability of the true class.

	AD	SQ	SCLC	CAR
AD	339	8	2	1
SQ	25	174	3	0
SCLC	10	1	32	0
CAR	4	2	0	38

Supplementary Table S2: Confusion matrix (based on leave-one-study-out cross-validation) for elastic net multinomial classifier at optimal value of regularization parameter. Rows correspond to the true class, columns correspond to the predicted class.

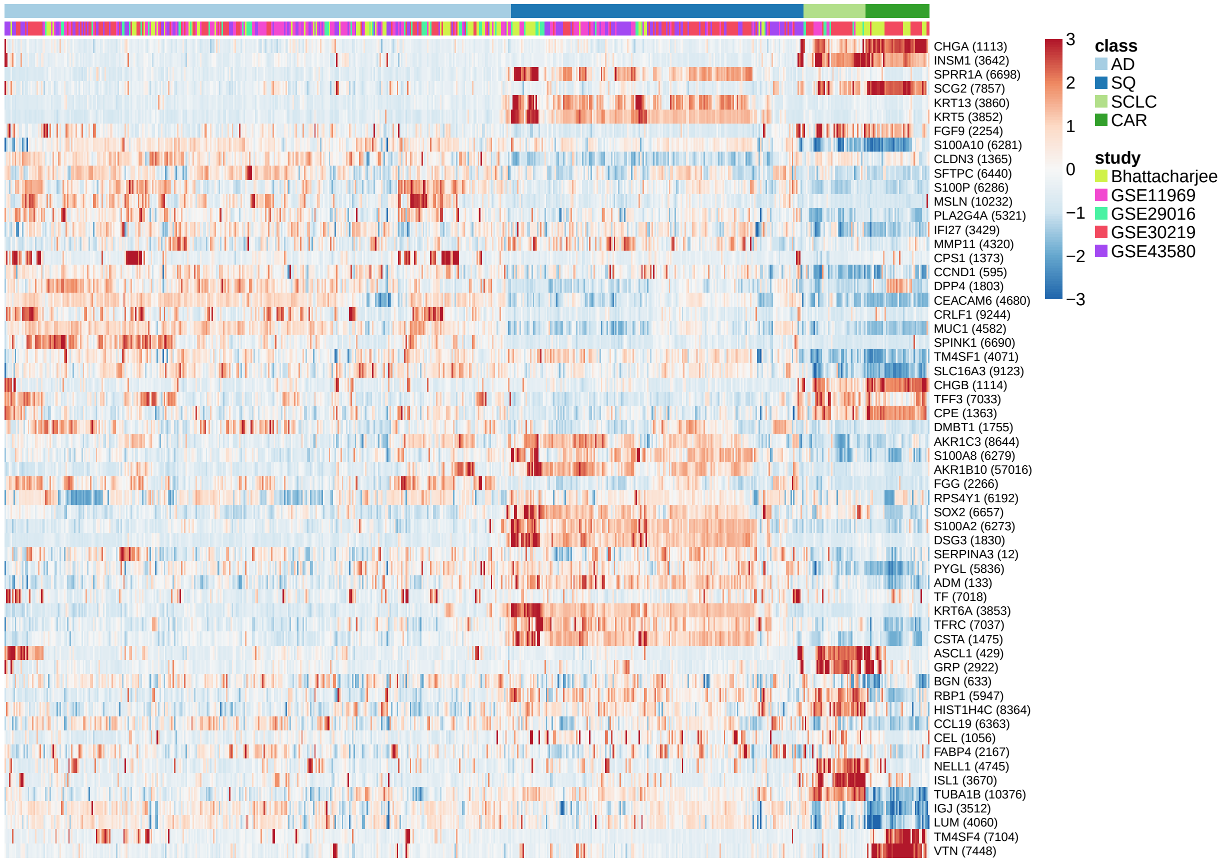

Supplementary Figure S3: Expression of genes in the multinomial classifier in samples from the discovery datasets. Expression values of each gene were standardized to mean 0 and standard deviation 1. Standardized expression values greater than 3 were set to 3, and values less than -3 were set to -3. Each row is a gene (Entrez Gene ID in parentheses), each column is a sample. The order of genes is the same as in Figure 2. Within each subtype, samples were ordered by hierarchical clustering.

	AD	SQ	SCLC	CAR
AD	361	15	6	2
SQ	27	202	2	1
SCLC	2	0	23	0
CAR	1	0	1	11

Supplementary Table S3: Confusion matrix on validation datasets for elastic net multinomial classifier trained on all discovery datasets. The value of the regularization parameter was determined by leave-one-study-out cross-validation. Rows correspond to the true class, columns correspond to the predicted class.

	AD	SQ	SCLC	CAR
AD	341	18	18	7
SQ	18	198	16	0
SCLC	1	0	24	0
CAR	0	0	2	11

Supplementary Table S4: Confusion matrix on validation datasets for elastic net multinomial classifier trained on only GSE30219. The value of the regularization parameter was determined by 5-fold cross-validation. Rows correspond to the true class, columns correspond to the predicted class.

	AD	SQ	SCLC	CAR
AD	373	4	0	7
SQ	125	104	2	1
SCLC	6	0	9	10
CAR	1	0	0	12

Supplementary Table S5: Confusion matrix on validation datasets for elastic net multinomial classifier trained on only the Bhattacharjee dataset. The value of the regularization parameter was determined by 5-fold cross-validation. Rows correspond to the true class, columns correspond to the predicted class.

	AD	SQ	SCLC	CAR
AD	333	9	1	7
SQ	23	175	1	3
SCLC	15	0	3	25
CAR	1	1	0	42

Supplementary Table S6: Confusion matrix (based on cross-validation) for PAM multinomial classifier trained using shrinkage parameter $\Delta = 12.6$, which results in a classifier containing approximately 100 genes. Rows correspond to the true class, columns correspond to the predicted class.

	AD	SQ	SCLC	CAR
AD	309	24	16	1
SQ	23	170	9	0
SCLC	5	0	36	2
CAR	2	0	1	41

Supplementary Table S7: Confusion matrix (based on cross-validation) for PAM multinomial classifier trained using shrinkage parameter $\Delta = 1.8$, which results in a classifier containing approximately 6000 genes. Rows correspond to the true class, columns correspond to the predicted class.

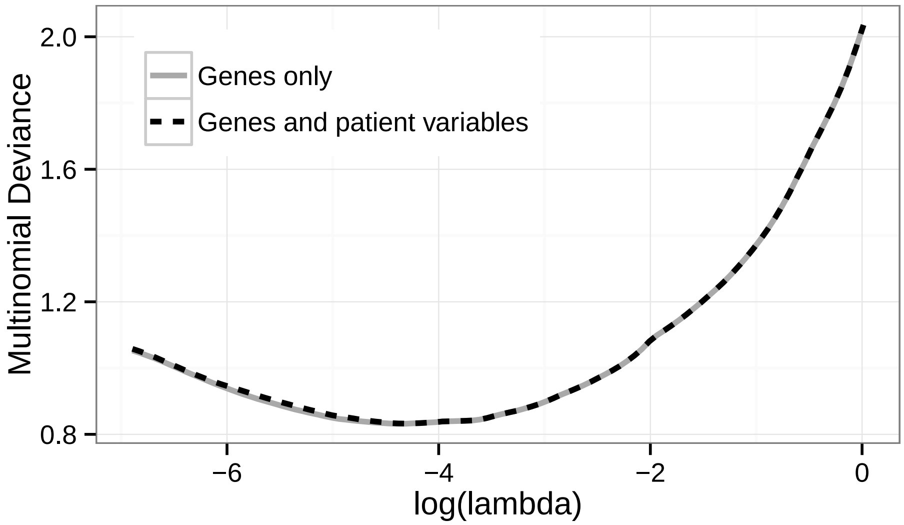

Supplementary Figure S4: Multinomial deviance as a function of the regularization parameter lambda for leave-one-study-out cross-validation, with and without patient sex, age, and smoking status.

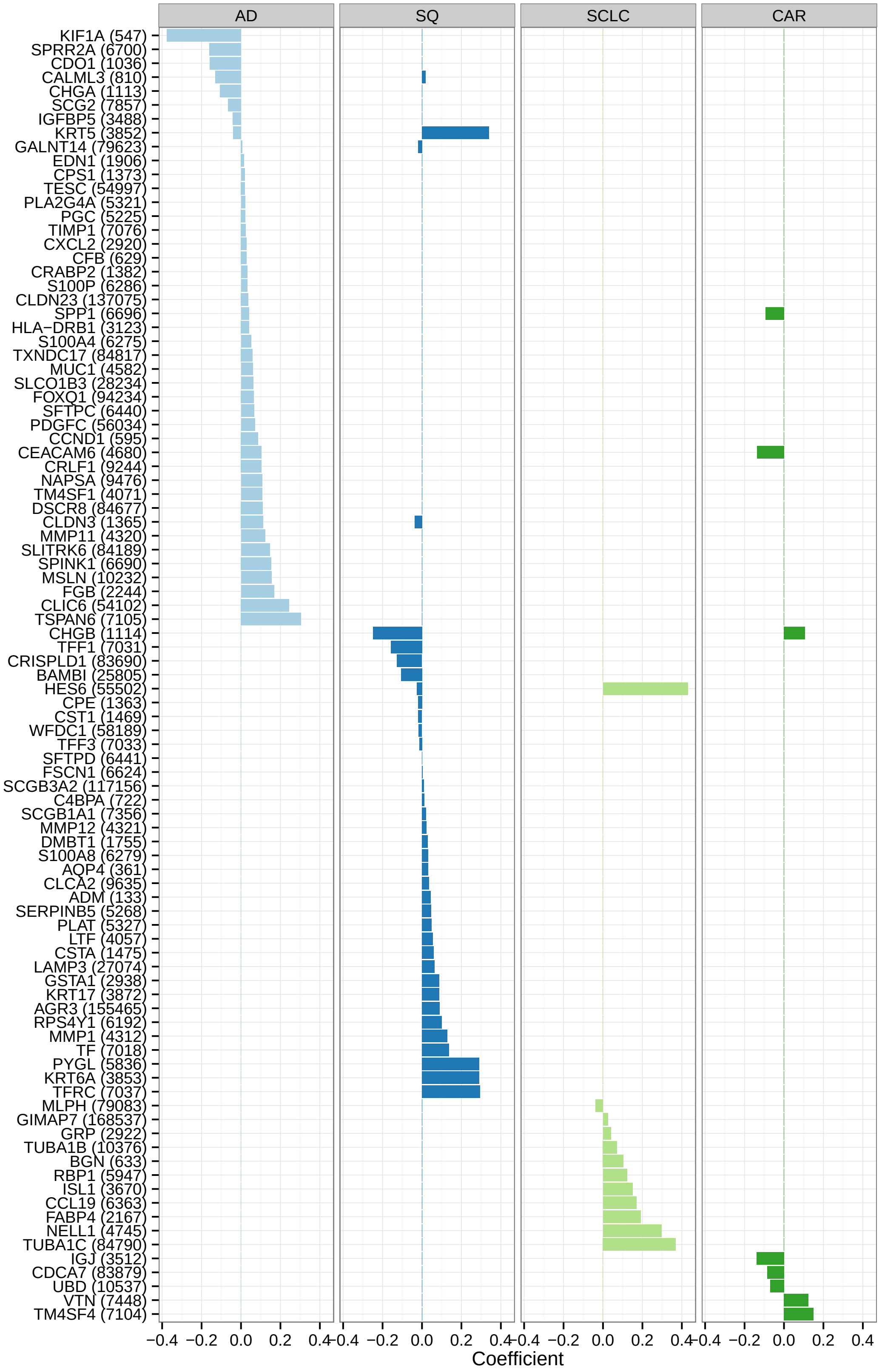

Supplementary Figure S5: Coefficients for multinomial classifier trained on the five datasets whose samples had corresponding patient sex, age, and smoking status. Patient variables and all genes were included. The regularization parameter used to train the model was the optimal one based on leave-one-study-out cross-validation.

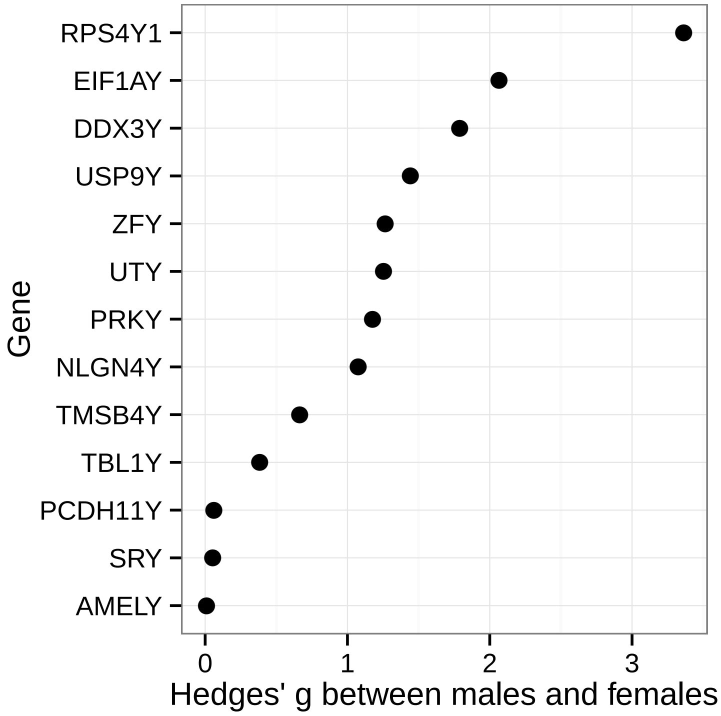

Supplementary Figure S6: Standardized difference of normalized expression between males and females, using Hedges' g, for each gene on the Y chromosome whose expression was measured on each of the five datasets shown in Figure 7.

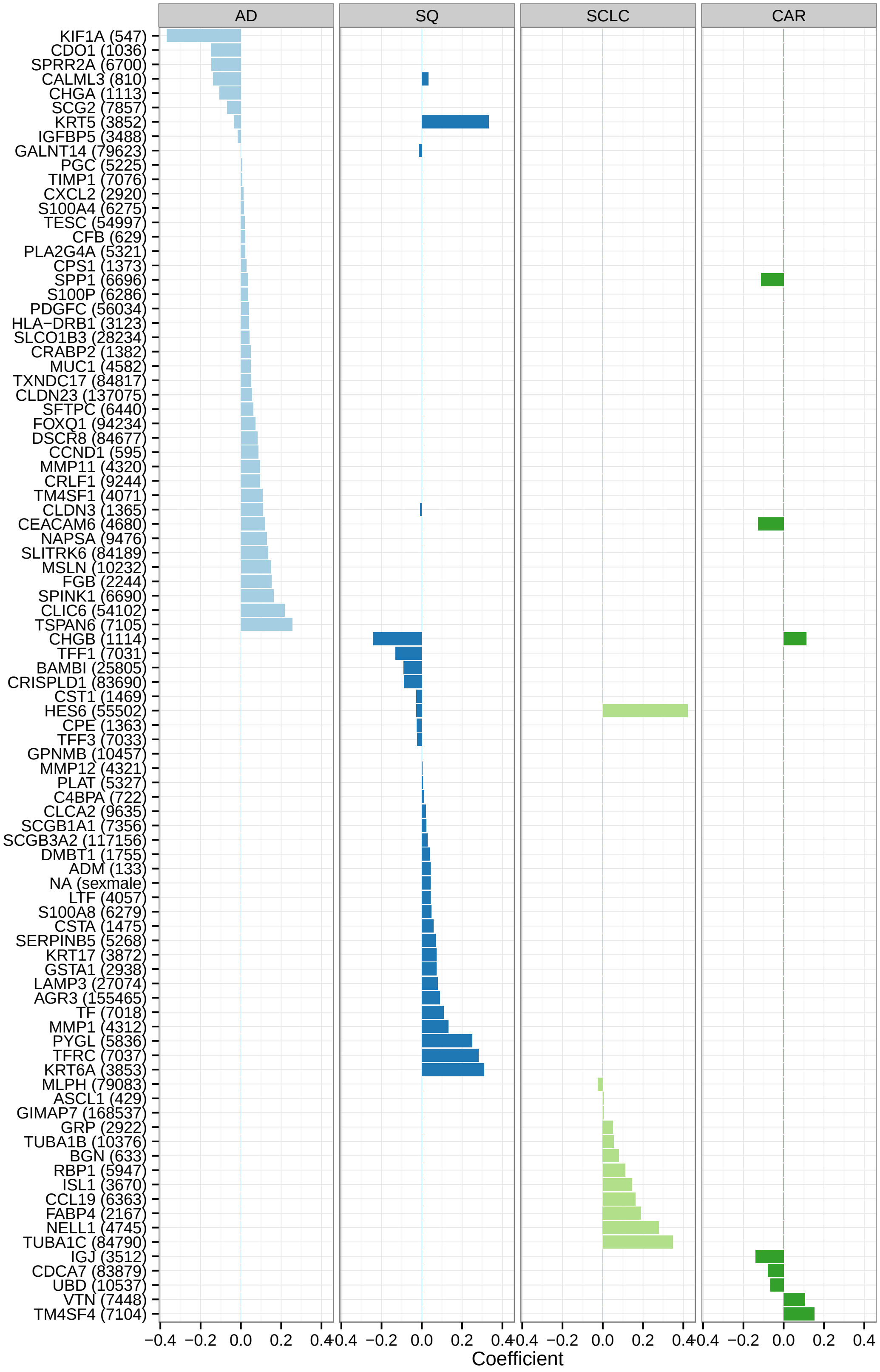

Supplementary Figure S7: Coefficients for multinomial classifier trained on the five datasets whose samples had patient sex, age, and smoking status. Patient variables were included, genes on the Y chromosome were excluded. The regularization parameter used to train the model was the optimal one based on leave-one-study-out cross-validation.
